# Trends of mental health care utilization among US adults from 1999 to 2018

**DOI:** 10.1186/s12888-023-05156-2

**Published:** 2023-09-12

**Authors:** Junzhe Wang, Yang Qiu, Xiaozhou Zhu

**Affiliations:** 1 Nanjing Medical University, Nanjing, 211166 China; 2 Jiangsu Provincial Academy of Environmental Science, Nanjing, 210036 China; 3 Jiangsu Key Lab of Environmental Engineering, Nanjing, 210036 China; 4 Department of Medical Insurance, The Affiliated Brain Hospital of Nanjing Medical University, Nanjing, 210029 China

**Keywords:** Mental health care, Trend, NHANES, Adult population

## Abstract

**Background:**

Mental health disorders affect millions of US adults, however, the trends and related factors for mental health care utilization in the US remain unknown.

**Aims:**

Our study aimed to assess the trend of mental health utilization and related socio-demographic factors in the US.

**Methods:**

The study included 55,052 individuals from the National Health and Nutrition Examination Survey (NHANES) in 1999–2018. Temporal trends in the percentages of mental health care utilizers were estimated across survey cycles. Trends and linked factors of mental health care utilization were assessed by a logistic regression model, while the non-linearity was estimated by restricted cubic splines.

**Results:**

From 1999 to 2018, the percentage of mental health care utilizers in the US adult population increased from 7.0 to 11.3% (*P* < 0.001); meanwhile, the trends in males and females were consistent. The percentage increased positively with age in individuals aged 20–39 (*P* < 0.001) or aged 60 and over (*P* = 0.003). The trends were consistent in three race/ethnicity groups (*P* < 0.05). The logistic regression analysis revealed that several disparities existed in the subpopulations. Older age, female, lower family poverty-income ratio (PIR), chronic diseases, higher educational level, and smoking were estimated to be associated with a higher percentage of mental health care.

**Conclusions:**

The percentage of mental health care utilizers took on an increasing trend in the US adult population from 1999 to 2018. These trends were also observed in the subpopulations, but with disparities. Future research for exploring factors associated with mental health care utilizations is necessary.

**Supplementary Information:**

The online version contains supplementary material available at 10.1186/s12888-023-05156-2.

## Introduction

The prevalence of mental disorders has kept increasing in the United States (US) and around the world. Approximately, 13.0% of the global population were estimated to have mild depression before the COVID-19 pandemic [1]. It was estimated that globally, the number of disability-adjusted life-years (DALYs) attributable to mental disorders was 125.3 million in 2019, while the percentages of mental disorder in total burden rose from 3.1 to 4.9% from 1990 to 2019 [2]. The prevalence of mental disorders is the highest in the US, posing a heavy burden on the health care system in this region, as indicated by a decreasing life expectancy and an increasing medical expenditure [2–4]. Considering their high prevalence and burden, the mental health care utilization should be analyzed, to better allocate medical services [5].

Health care utilization can reflect the demand for, the cost of medical services for certain diseases (such as depression or anxiety) [6, 7]. This indicator can be used to guide the allocation of medical services. An effective health care utilization can benefit the early identification of mental disorders, prolong patients’ life expectancy, and improve the quality of life [8, 9]. Although mental health care utilization in US sub populations have been reported, however, the latest statistics (after 2015) and factors associated with mental health care utilizations have not been well characterized, especially in US general adults, which should be further investigated [10–12].

Here, we performed a cross-sectional analysis for estimating the temporal trends in the percentage of mental health care utilizers in the general US population from 1999 to 2018. We also assessed the trends in subpopulations stratified with age, race/ethnicity, and sex. Furthermore, we estimated the associations between baseline factors and health care utilization using a survey-weighted generalized linear regression model.

## Methods

### Study design and population

This cross-sectional study included 55,052 individuals from the National Health and Nutrition Examination Survey (NHANES). NHANES is a survey in which participants are recruited through multiple-stage sampling and applied complex survey design to obtain demographic data from noninstitutionalized civilian population in the US [13]. From 1999 to 2018, a total of 10 cycles of data had been collected into the NHANES. The research protocol had passed the ethics review and all participants had provided signed informed consent. The detailed information for NHANES could be seen on the website [13]. For this study, the population was restricted to those aged 20 years or over. Participants with complete data of sociodemographic characteristics and mental health care utilization were included. The data analysis was conducted from March 4, 2022 to May 14, 2023.

### Outcomes

The main outcome was the percentage of mental health care utilizers (those who seek for medical services for mental disorders), which was calculated according to the data collected through the question “During the past 12 months, (have you/has SP) seen or talked to a mental health professional such as a psychologist, psychiatrist, psychiatric nurse, or clinical social worker about (your/his/her) health?”

### Sociodemographic factors

Sociodemographic factors, including age, sex, race/ethnicity, body mass index (BMI), poverty-income ratio (PIR), marital status, self-reported diseases, educational level, and current smoking status, were analyzed. Age, sex, marital status, PIR, educational level, self-reported diseases, and race/ethnicity were collected through standardized questionnaire in the interview [13]. Age was coded as 20–39, 40–59, and ≥ 60 years. Race/ethnicity was recoded into four categories as Non-Hispanic White, Hispanic, Non-Hispanic Black, and non-Hispanic others (including Asians). PIR is the ratio of family income to poverty; The Department of Health and Human Services (HHS) poverty guidelines were used as the poverty measure to calculate this ratio, the variable was categorized as < 1.3, 1.3–3.5, and > 3.5 [14]. Marital status was coded as married (including living with partner), divorced, widowed, and never married. BMI was calculated through dividing weight in kilograms by height in meters squared, while both weight and height were measured in the mobile examination center (MEC). BMI was categorized into < 25 kg/m^2^, 25-29.9 kg/m^2^, and ≥ 30 kg/m^2^, as previously reported [15]. Self-reported diseases were determined from the questionnaire about the history of malignant cancer, coronary heart disease, congestive heart failure, angina, stroke, heart attack, liver condition, chronic bronchitis, and emphysema. Smoking status was determined with the question “Have you smoked at least 100 cigarettes in your entire life?” by the NHANES interview.

### Statistical analysis

All the analysis accounted for the complex design and used appropriate weights, strata, and primary sampling units (PSU) as feasible [16, 17]. Unweighted demographic characteristics were displayed to make better understanding for the distribution of the variables. We estimated the weighted percentage (95%CI) of mental health utilizers. Age, sex, and race/ethnicity were used to adjust our models. We also performed a subgroup analysis according to race/ethnicity (non-Hispanic white: NHW, non-Hispanic black: NHB, Hispanic and non-Hispanic others) or age (20–39 years, 40–59 years, 60 years or over). We included Asians in the Non-Hispanic Other race and ethnicity category for the stratified analyses.

A logistic regression analysis was applied to estimate the linear relationships of mental health care utilization with various factors [18]. We adjusted age, sex, and race/ethnicity in the models as previous recommended [19]. We also estimated the non-linearity of the trends by establishing cubic spline models, with knots set at 3 following the previously published article [20]. A sensitivity analysis was performed via adjusting family PIR and educational level into the models.

A logistic regression model was established incorporating age, race/ethnicity, sex, family PIR, marital status, BMI, self-reported non-communicable diseases (NCDs), current smoking status and educational level [21]. We did not conduct multiple comparison, considering that the results were exploratory and type I error would happen in our models.

All analyses were performed using R 4.0.1. *survey* 4.0 packages were utilized for population-based estimation of the results. Two-sided *P* value < 0 0.05 was considered statistically significant.

## Results

### Demographic characteristics

The demographic characteristics are shown in Table 1. A total of 55,052 individuals were included in the cross-sectional study from 1990 (N = 4869) to 2018 (N = 5568), including 26,457 males and 28,595 females, with a mean age of 50.52 in 1999 and 51.50 in 2018. Generally, the non-Hispanic White made up the highest percentage in our analysis, 45.5% in 1999 and 34.8% in 2018.

### Percentages of mental health care utilizers in the US

**Fig. 1:**
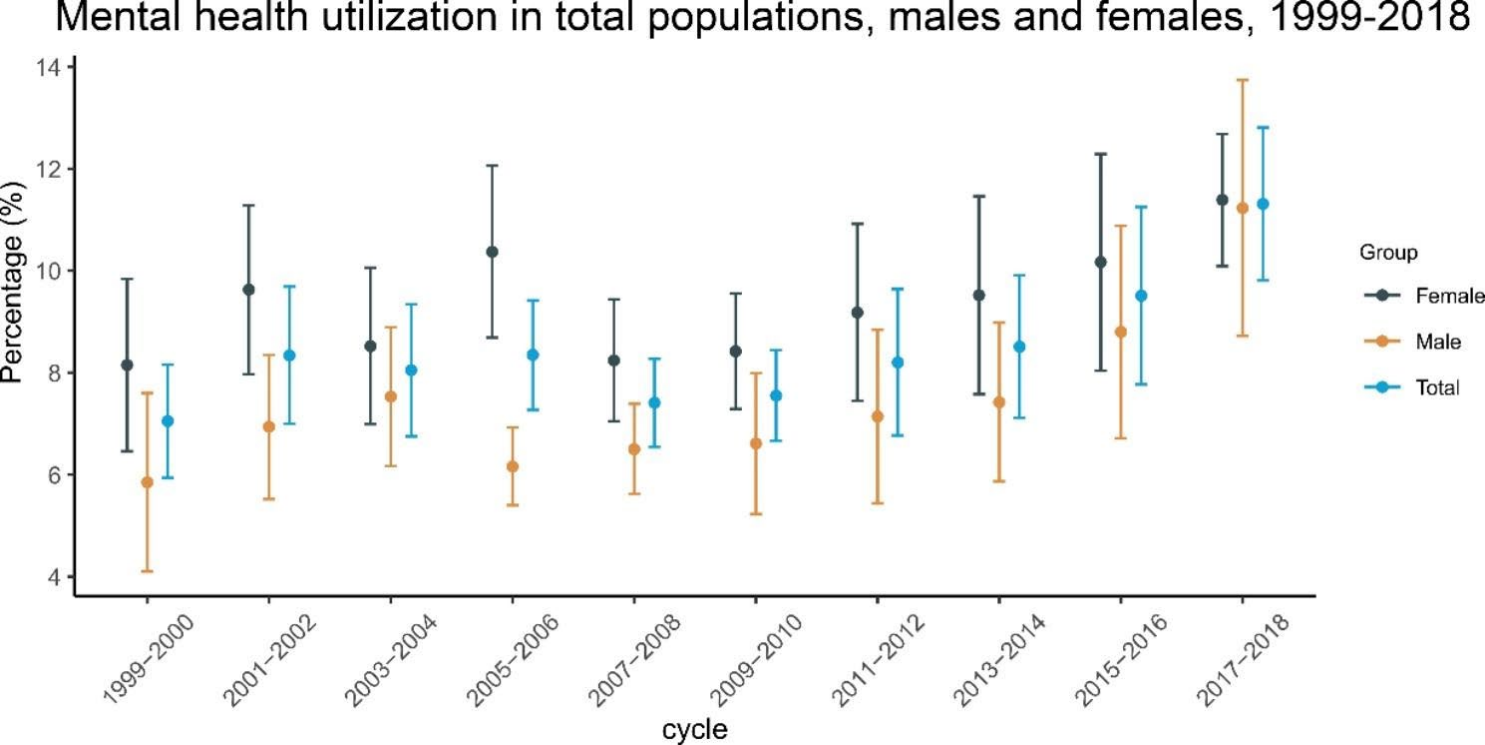
Trends of mental health care utilization in the total population, males and females, 1999–2018

**Fig. 2:**
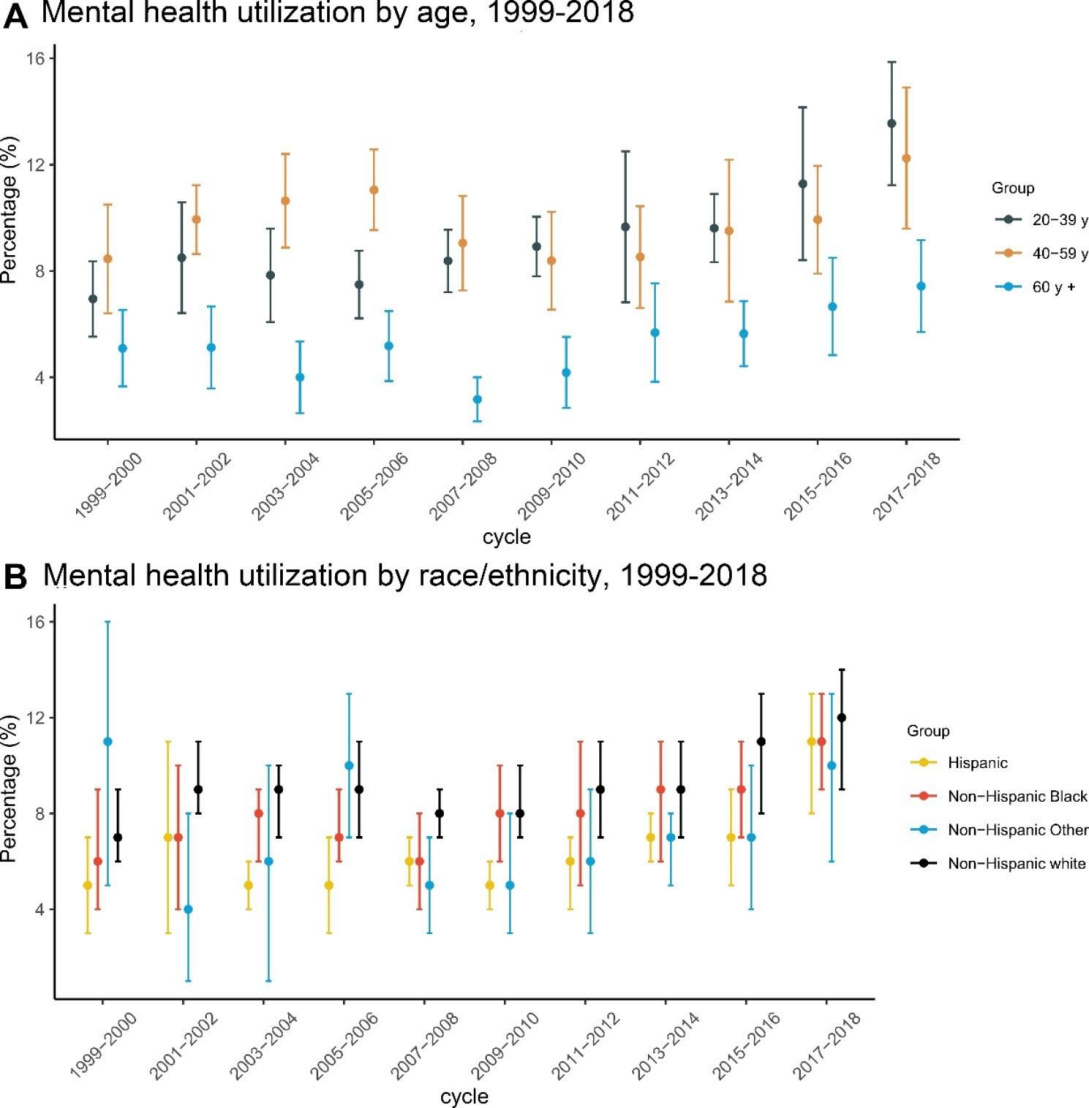
Trends of mental health care utilization in subpopulations stratified by age and race/ethnicity, 1999–2018. **A** Mental health care utilization by age, 1999–2018. **B** Mental health care utilization by race/ethnicity, 1999–2018

The percentages of mental health care utilizers during different study cycles in the general US population are shown in Fig. 1 and TableS1, and the estimated trends are shown in Fig. 2. Briefly, in the adult population, the percentage of mental health care utilizers increased from 7.0% (95%CI: 5.9-8.2%) in 1999–2000 to 11.3% (95%CI: 9.8-12.8%) in 2017–2018. A consistent trend was also observed from 1999 to 2018. The percentage increased by an average of 5% (95%CI: 3-7%) per survey cycle. In subgroups stratified by sex, we also observed that the percentage increased by 7% (95%CI: 4-10%) per survey cycle in males and 3% (95%CI: 1-6%) in females. The sex-specific percentages are shown in Table S2. We found that in 1999–2000, the percentage was 5.9% (95%CI: 4.1-7.6%) in males and 8.2% (95%CI: 6.5-9.8%) in females, while in 2017–2018, the percentage was 11.2% (95%CI: 8.7-13.7%) in males and 11.4% (95%CI: 10.1-12.7%) in females.

### Age- and race/ethnicity-specific percentages of mental health care utilizers in the US

**Fig. 3:**
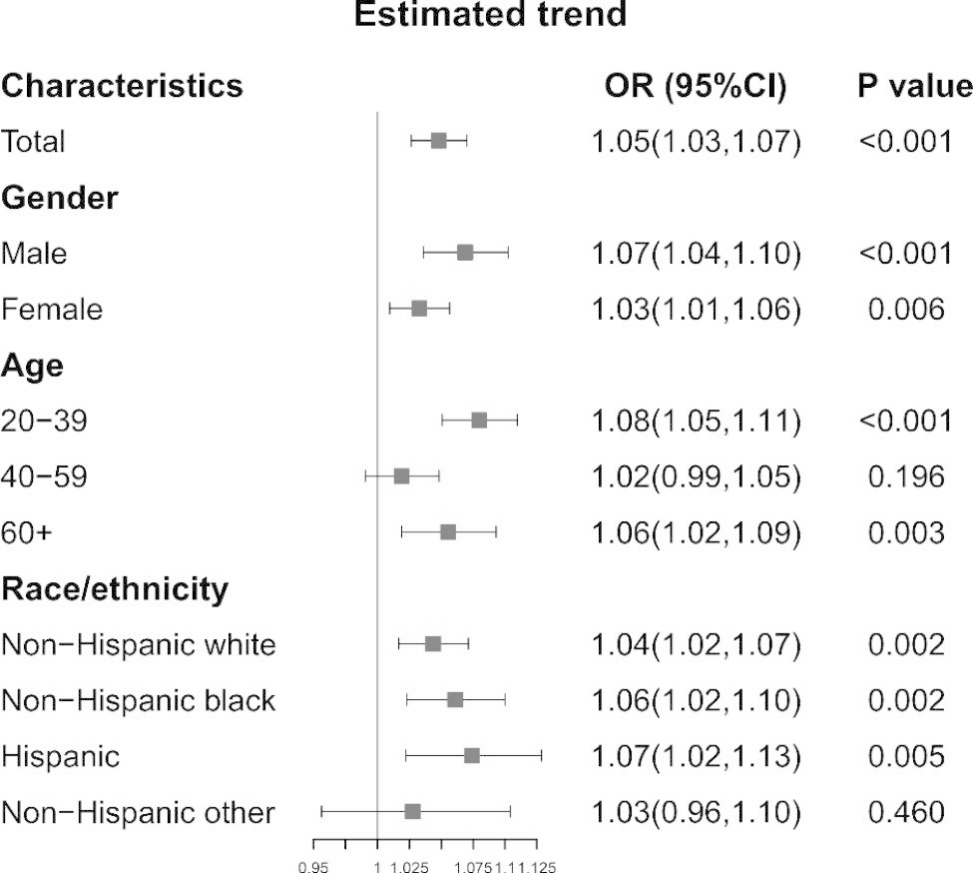
Estimated trends of mental health care utilization in the adult population and subpopulations, 1999–2018. Models were adjusted with age, sex, and race/ethnicity

Next, we assessed the age- and race/ethnicity-specific percentages of mental health care utilizers. As shown in Figs. 2A and 3, we found the increasing trend of the percentage was more significant in individuals aged 20–39 years and individuals aged 60 years or over (*P* for individuals aged 20–39 years < 0.001; *P* for individuals aged 60 years or over = 0.003). In individuals aged 40–59 years, no significant increasing trend was found. Interestingly, we found that the percentage peaked in 2007–2008 among individuals aged 40–59 years (9.1%, 95%CI: 7.2-10.8%), in 2017–2018 among individuals aged 20–39 years (13.5%, 95%CI: 11.2-15.9%) (Table S2).

As displayed in Figs. 2B and 3, we found that the percentage increased most significantly among NHW individuals from 2001 to 2018 (prevalence in 2001–2002: 7.4%, 95%CI: 5.9-8.9%; prevalence in 2017–2018: 11.7%, 95%CI: 9.3-14.2%). Briefly, the increasing trends in NHW, NHB and Hispanic individuals were all significant from 1999 to 2018 (NHW: 4%, 95%CI: 2-7%, P = 0.002; NHB: 6%, 95%CI: 2-10%, P = 0.002; Hispanic: 7.2%, 95%CI: 2.2-12.1%, P = 0.005; Table S3).

The results of sensitivity analysis were consistent with those in the main analysis, indicating the robustness of our analyses (Table S4). In addition, we found the estimated trend attenuated after adjusting for socio-economic status.

**Fig. 4:**
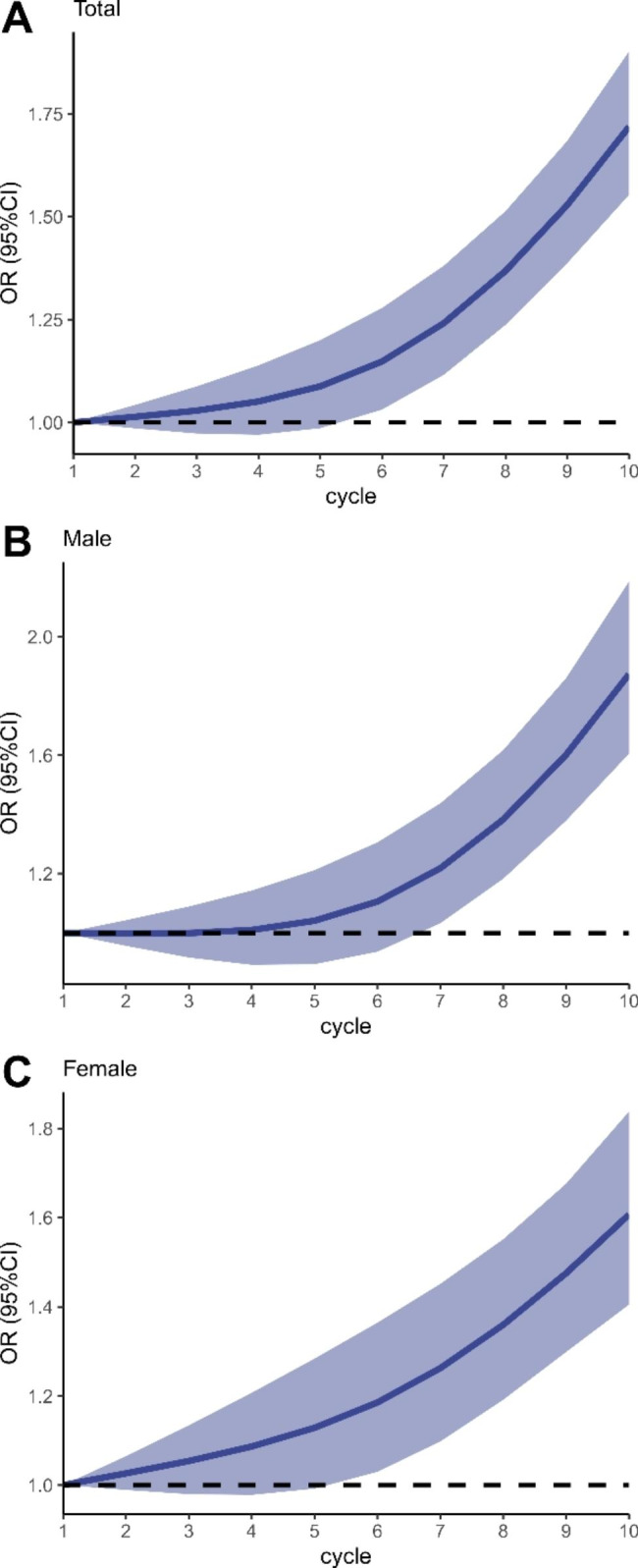
Non-linear associations between survey cycle and mental health care utilization in the adult population, 1999–2018. Models were adjusted with age, sex, and race/ethnicity. Cycle was coded from 1–10 to represent survey year from 1999–2000 to 2017–2018. **(A)** Non-linear trends for mental health care utilization in total populations. **(B)** Non-linear trends for mental health care utilization in Males. **(C)** Non-linear trends for mental health care utilization in females. P for non-linearity in total, males and females is < 0.0001, < 0.0001, and 0.1163, respectively

We also determined the non-linearity of the trend in the adult population and subpopulations. As shown in Fig. 4 and results of sensitivity FigureS1, we found that in the adult population and males, a non-linear trend of mental health care utilization was observed (P for non-linearity in the total population < 0.0001, P for non-linearity in males < 0.0001), and the non-linear trend in NHB populations was also noted (P for non-linearity = 0.0093). In elders, a significant U-shaped trend showed up (P for non-linearity < 0.0001).

### Factors linked to mental health care utilization

**Fig. 5:**
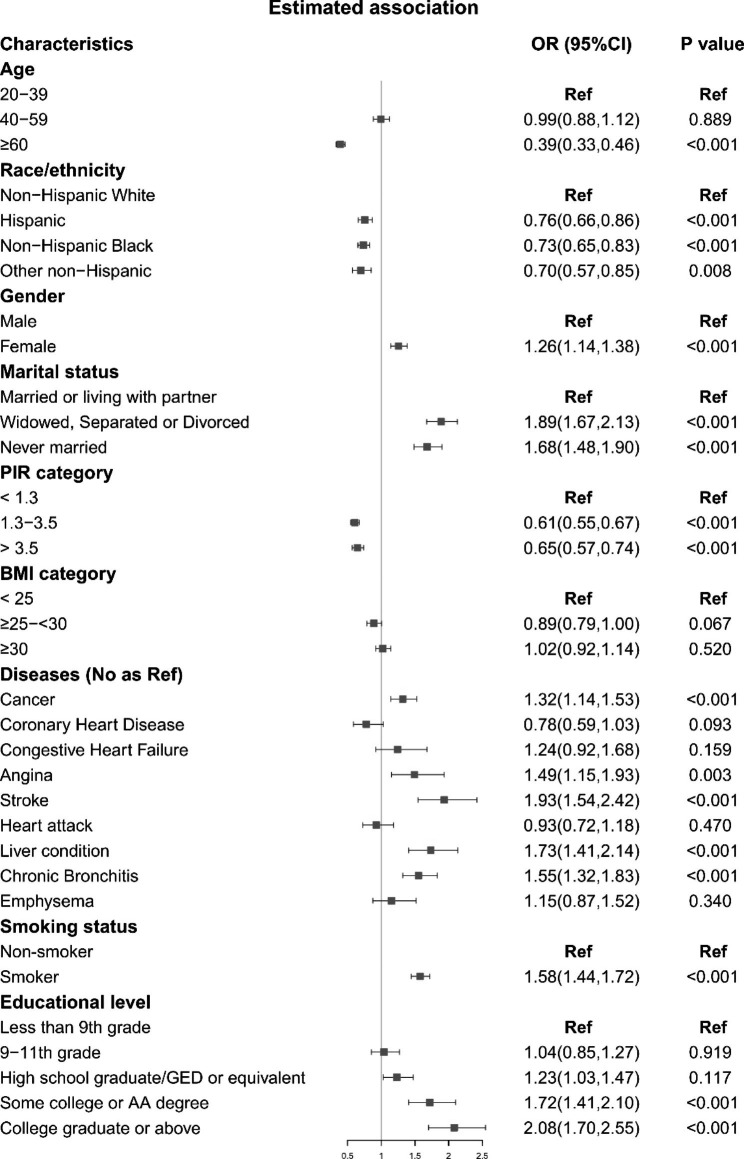
Associations between sociodemographic characteristics and mental health care utilization in the adult population, 1999–2018. PIR: family poverty-income ratio; BMI: body mass index; GED: General education degree; AA: Associate of Arts

Next, we estimated associations between demographic characteristics and health care utilization in the US during 1999–2018 (Fig. 5). We found that age ≥ 60 years (OR for age ≥ 60 years vs. age 20–39: 0.39, 95%CI: 0.33–0.46, P < 0.001), higher PIR (OR for PIR 1.3–3.5 vs. PIR < 1.3: 0.61, 95%CI: 0.55–0.67, P < 0.001; OR for PIR > 3.5 vs. PIR < 1.3: 0.65, 95%CI: 0.57–0.74, P < 0.001), non-NHW were factors associated with a lower percentage of mental health care utilizers (OR for Hispanic vs. NHW: 0.76, 95%CI: 0.66–0.86, P < 0.001; OR for NHB vs. NHW: 0.73, 95%CI: 0.65–0.83, P < 0.001; OR for non-Hispanic others vs. NHW: 0.70. 95%CI: 0.57–0.85, P = 0.008).

Besides, we also found that female, unmarried, smoking and higher educational level were factors associated with an increased mental health care utilization (OR for female vs. male: 1.26, 95%CI: 1.14–1.38, P < 0.001; OR for smoker vs. non-smoker: 1.58, 95%CI: 1.44–1.72, P < 0.001; OR for widowed, separated or divorced vs. married: 1.89, 95%CI: 1.67–2.13, P < 0.001; OR for never married vs. unmarried: 1.68, 95%CI: 1.48–1.90, P < 0.001; OR for college vs. less than 9th grade: 2.08, 95%CI: 1.70–2.55, P < 0.001; OR for college graduate vs. less than 9th grade: 1.72, 95%CI: 1.41–2.10, P < 0.001). For NCDs, we found that individuals with cancer (OR: 1.32, 95%CI: 1.14–1.53, P < 0.001), angina (OR: 1.49, 95%CI: 1.15–1.93, P = 0.003), stroke (OR: 1.93, 95%CI: 1.54–2.42, P < 0.001), liver disorder (OR: 1.73, 95%CI: 1.41–2.14, P < 0.001), chronic bronchitis (OR: 1.55, 95%CI: 1.32–1.83, P < 0.001) had a higher odds to utilize mental health service than the participants without such diseases.

## Discussion

### Overview of our findings

Our study enrolled the data of 55,052 participants from a national cross-sectional study and examined the temporal trends of mental health care utilization in the adult population and subpopulations. In the adult population, we found that the percentage of mental health care utilizers showed an increasing trend from 1999 to 2018. This increasing trend was also observed in each subgroup, but also with differences from each other. In the explorative analysis, we found that female, unmarried, smoking, higher educational level, diagnosed NCDs were positively, while age ≥ 60 years, higher PIR, and non-NHW were negatively associated with the percentage of mental health care utilizers in the past decades (1999–2018).

### An increasing trend of mental health utilization in the general US population

The percentage of mental health care utilizers in the general US increased trend from 7.0% to 1999 to 13.1% in 2018. This increase may also suggest that the increasing prevalence of mental disorders and demand for psychological care in the recent years [22, 23]. This finding provides evidence that related health care policy about mental disorders should be upgraded. Notably, in sub-populations, this monotonic upward trend was also obvious, suggesting that the mental health care utilization has undergone a fundamental change over the past decades. During the COVID-19 pandemic, the prevalence of depression or other mental disorders has substantially increased, so has the demand for mental health care [24]. In our study, we found that the mental health care utilization had already rose before the pandemic. Then, we speculate that COVID-19 might have aggravated the situation, and health care policies should be adjusted to meet the demand for mental health care, especially early diagnosis, and intervention in the US. In the sensitivity analysis, we found that adjusted educational level may attenuate the increasing trend, this suggested that socio-economic position may be an important modifier for the trends of mental health care utilization. Hence, it is necessary to clarify the role of socio-economic position on mental health care utilization in the future studies.

In the analysis, we also found that a significant increment of mental health care utilization in most recent years (2017–2018) than the previous survey cycles. Two reasons may be associated with the results we observed. First, the mental health disorder had a relatively higher prevalence in 2017–2018, which was determined by previous studies [25, 26]. The increasing trend suggested that the demands might increase in the most recent survey cycles. Second, the sub-analyses had relatively smaller sample size, which determined that the statistical power may be lower. Therefore, the reasons for the increasing mental health care utilization in most recent years should be further investigated. Taken together, the estimated trend in several populations may be caused by increment in recent years other than long-term shift of mental health care utilization, which should be considered carefully.

### Disparity in mental health care utilization among subpopulations

In the stratified analysis, we found the significant disparities in the percentages of mental health care utilizers between subpopulations. Briefly, females had a higher percentage than males, and the young had a higher percentage than the middle-aged and older. This may be possibly caused by the different socio-economic statuses (SESs) between subpopulations [27, 28]. Previous research has indicated that lower SES may have less access to high-quality health care, thereby bringing an inequality in health care utilization between populations [29]. In terms of race/ethnicity, the prevalence of mental disorders has increased in the NHW subpopulation and some other minority groups [30, 31]. As expected, our study found that the percentage was the highest in the NHW subpopulation from 1999 to 2018. Therefore, efforts should be taken to provide equal health care to populations with different race/ethnicity or SESs and reduce the disease burden.

In the subpopulation analysis, we found that the increasing trend was the most significant in the recent survey cycle (2017–2018). Previous studies have indicated that the prevalence of depression increased significantly from 5.4% in 2005–2006 to 8.7% in 2017–2018, which may partially explain the highest mental health care utilization we observed [25, 26].

### Explorative analysis

The explorative analysis suggested that several demographic characteristics were associated with mental health care utilization. Age ≥ 60 years, higher PIR, and non-NHW were negatively related to mental health care utilization. These associations were also consistent in race/ethnicity. The reasons may be that individuals with higher PIR and age are likely to have higher SES, quality of life and self-satisfaction, which may reduce the requirements for mental health care [27, 32, 33]. Meanwhile, female, unmarried, smoking, higher educational level, diagnosed NCDs were positively associated with mental health utilization in 2017–2018, which suggested that these might be factors increasing the demand for mental health care. Previous studies have found that unhealthy lifestyle, unmarried status, and NCDs can raise the risk of developing mental disorder, depression, and stress, which may explain the associations we estimated [34, 35]. Another possible reason is that in individuals with NCDs or other diseases, pain may cause psychological disorders, which drive them to seek for mental health care [33, 36]. Specifically, we found that in females and middle-aged participants, the increasing trend in mental health utilization was insignificant after adjustment for SES, suggesting that SES may powerfully mediate the effects of sex and age. Future studies should be performed to analyze the demand for mental health care in those with different SES levels.

### Comparison with previous studies

Previous studies have estimated the percentages of mental health care utilization in several sub-populations, such as children and young adults. A study based on data from survey come from > 350,000 students at 373 campuses that participated in the Healthy Minds Study showed that in college students, prevalence of mental health care utilization has increased over the past decades [37]. Another study also determined that children and adolescents in US have increased prevalence of mental health care utilization [12]. Taken together, the previous studies focused on young adults, children, and adolescents, in which the reported results in these studies were consistent with those in our studies. Beyond the evidence, our studies characterized the percentages of mental health care utilization in several sub-populations in US and provided population-representative estimations. In previous studies, marital status, gender, and educational level were found to be associated with mental health care utilization [38, 39]. In our studies, the results were similar, and the studies revealed several new findings as comorbidities, unhealthy lifestyles (smoking), and lower income were associated with mental health care utilization, which is not reported in previous studies.

### Strengths and limitations

Our study has several strengths. First, the study population was collected from the NHANES that recruits samples through complex and multi-stage methods; therefore, the estimated mental health care utilization could represent that in the adult population in the US [40]. Second, our data from 1999 to 2018 provided an overview on the trend of mental health care utilization in the past 20 years.

Our study has several limitations. First, the reported mental health care utilization was only determined by questionnaire in a single time, which might be misclassified in our study. Second, only a cross-sectional study was applied to explore the associations between demographic characteristics and mental health care utilization; however, a cohort study should be further performed to validate the causal associations [41]. Third, the results were based on the data from a general US population, while the geographical disparities among states and counties were not analyzed [42]. Fourth, the trends of mental health care utilization are estimated by logistic regression models, in which the significance were derived from statistical analysis. The trend we observed should be further validated by real-world observations.

## Conclusion

Generally, mental health care utilization in the US showed an increasing trend from 1999 to 2018. These trends were also observed in the subpopulations, but with disparities. Future research for exploring factors associated with mental health care utilizations is necessary.

**Table 1:** Demographic characteristics of the study population

Characteristics ^a^	Study cycle									
	1999–2000	2001–2002	2003–2004	2005–2006	2007–2008	2009–2010	2011–2012	2013–2014	2015–2016	2017–2018
	N = 4869	N = 5409	N = 5038	N = 4975	N = 5935	N = 6217	N = 5558	N = 5767	N = 5716	N = 5568
**Mental healthcare utilization (%)**										
No	4567 (93.8)	5041 (93.2)	4678 (92.9)	4610 (92.7)	5529 (93.2)	5757 (92.6)	5124 (92.2)	5284 (91.6)	5217 (91.3)	4996 (89.7)
Yes	302 (6.2)	368 (6.8)	360 (7.1)	365 (7.3)	406 (6.8)	460 (7.4)	434 (7.8)	483 (8.4)	499 (8.7)	572 (10.3)
**Age (%)**										
20–39	1692 (34.8)	1925 (35.6)	1741 (34.6)	1922 (38.6)	1910 (32.2)	2083 (33.5)	1957 (35.2)	1954 (33.9)	1952 (34.1)	1687 (30.3)
40–59	1349 (27.7)	1613 (29.8)	1398 (27.7)	1484 (29.8)	1871 (31.5)	2062 (33.2)	1811 (32.6)	1973 (34.2)	1865 (32.6)	1731 (31.1)
≥ 60	1828 (37.5)	1871 (34.6)	1899 (37.7)	1569 (31.5)	2154 (36.3)	2072 (33.3)	1790 (32.2)	1840 (31.9)	1899 (33.2)	2150 (38.6)
**Race/ethnicity (%)**										
Non-Hispanic White	2213 (45.5)	2856 (52.8)	2688 (53.4)	2492 (50.1)	2761 (46.5)	2976 (47.9)	2040 (36.7)	2471 (42.8)	1863 (32.6)	1935 (34.8)
Hispanic	1590 (32.7)	1350 (25.0)	1137 (22.6)	1157 (23.3)	1699 (28.6)	1771 (28.5)	1118 (20.1)	1275 (22.1)	1760 (30.8)	1251 (22.5)
Non-Hispanic Black	905 (18.6)	1012 (18.7)	993 (19.7)	1122 (22.6)	1227 (20.7)	1122 (18.0)	1454 (26.2)	1177 (20.4)	1198 (21.0)	1298 (23.3)
Non-Hispanic others	161 (3.3)	191 (3.5)	220 (4.4)	204 (4.1)	248 (4.2)	348 (5.6)	946 (17.0)	844 (14.6)	895 (15.7)	1084 (19.5)
**Sex (%)**										
Male	2263 (46.5)	2535 (46.9)	2417 (48.0)	2386 (48.0)	2910 (49.0)	3005 (48.3)	2738 (49.3)	2757 (47.8)	2744 (48.0)	2702 (48.5)
Female	2606 (53.5)	2874 (53.1)	2621 (52.0)	2589 (52.0)	3025 (51.0)	3212 (51.7)	2820 (50.7)	3010 (52.2)	2972 (52.0)	2866 (51.5)
**Marital status (%)**										
Married	2440 (56.3)	3045 (56.3)	2689 (53.4)	2687 (54.0)	3116 (52.5)	3186 (51.2)	2682 (48.3)	2964 (51.4)	2886 (50.5)	2736 (49.1)
Widowed	473 (10.9)	610 (11.3)	587 (11.7)	461 (9.3)	562 (9.5)	559 (9.0)	467 (8.4)	436 (7.6)	421 (7.4)	462 (8.3)
Divorced	374 (8.6)	453 (8.4)	472 (9.4)	470 (9.4)	657 (11.1)	679 (10.9)	571 (10.3)	658 (11.4)	613 (10.7)	641 (11.5)
Separated	173 (4.0)	168 (3.1)	133 (2.6)	153 (3.1)	203 (3.4)	207 (3.3)	203 (3.7)	177 (3.1)	190 (3.3)	202 (3.6)
Never married	674 (15.5)	824 (15.2)	851 (16.9)	789 (15.9)	992 (16.7)	1099 (17.7)	1188 (21.4)	1112 (19.3)	1048 (18.3)	1006 (18.1)
living with partner	193 (4.5)	301 (5.6)	303 (6.0)	408 (8.2)	401 (6.8)	483 (7.8)	440 (7.9)	417 (7.2)	555 (9.7)	515 (9.2)
**PIR category (%)**										
< 1.3	1277 (31.0)	1353 (27.2)	1348 (28.5)	1238 (26.2)	1651 (30.7)	1886 (33.7)	1834 (36.2)	1840 (34.6)	1644 (32.4)	1348 (28.2)
1.3–3.5	1571 (38.1)	1922 (38.6)	1904 (40.3)	1880 (39.8)	2105 (39.2)	2109 (37.7)	1714 (33.8)	1836 (34.6)	2012 (39.6)	1988 (41.6)
> 3.5	1278 (31.0)	1698 (34.1)	1470 (31.1)	1605 (34.0)	1617 (30.1)	1599 (28.6)	1516 (29.9)	1636 (30.8)	1423 (28.0)	1442 (30.2)
**BMI category (%)**										
< 25	1407 (32.2)	1505 (32.1)	1478 (31.8)	1427 (30.5)	1625 (29.0)	1682 (28.1)	1680 (32.1)	1668 (30.2)	1485 (27.5)	1337 (25.8)
≥ 25 - <30	1539 (35.2)	1752 (37.4)	1629 (35.1)	1603 (34.3)	1933 (34.5)	2027 (33.8)	1683 (32.1)	1768 (32.0)	1732 (32.1)	1667 (32.2)
≥ 30	1420 (32.5)	1431 (30.5)	1537 (33.1)	1647 (35.2)	2049 (36.5)	2285 (38.1)	1873 (35.8)	2082 (37.7)	2186 (40.5)	2170 (41.9)
**Cancer (%)**										
No	4479 (92.1)	4882 (90.4)	4549 (90.5)	4557 (91.7)	5348 (90.3)	5590 (90.0)	5065 (91.2)	5220 (90.5)	5162 (90.4)	4978 (89.4)
Yes	386 (7.9)	519 (9.6)	477 (9.5)	414 (8.3)	576 (9.7)	620 (10.0)	488 (8.8)	547 (9.5)	549 (9.6)	588 (10.6)
**Coronary heart disease (%)**										
No	4634 (95.8)	5129 (95.5)	4746 (94.7)	4749 (96.0)	5653 (95.7)	5936 (95.9)	5341 (96.5)	5517 (96.0)	5446 (95.7)	5287 (95.2)
Yes	203 (4.2)	240 (4.5)	263 (5.3)	200 (4.0)	253 (4.3)	253 (4.1)	196 (3.5)	232 (4.0)	244 (4.3)	265 (4.8)
**Congestive heart failure (%)**										
No	4679 (96.6)	5198 (96.5)	4820 (96.1)	4779 (96.4)	5693 (96.3)	6024 (97.2)	5356 (96.6)	5577 (96.8)	5491 (96.2)	5350 (96.4)
Yes	167 (3.4)	187 (3.5)	196 (3.9)	179 (3.6)	217 (3.7)	174 (2.8)	187 (3.4)	182 (3.2)	214 (3.8)	201 (3.6)
**Angina (%)**										
No	4665 (96.3)	5178 (96.3)	4807 (95.8)	4803 (96.9)	5749 (97.2)	6044 (97.5)	5411 (97.7)	5623 (97.6)	5565 (97.7)	5377 (97.1)
Yes	179 (3.7)	199 (3.7)	212 (4.2)	155 (3.1)	164 (2.8)	155 (2.5)	129 (2.3)	136 (2.4)	133 (2.3)	161 (2.9)
**Stroke (%)**										
No	4678 (96.1)	5195 (96.2)	4819 (95.8)	4773 (96.1)	5659 (95.6)	5982 (96.3)	5326 (95.9)	5560 (96.5)	5502 (96.3)	5285 (95.1)
Yes	188 (3.9)	204 (3.8)	211 (4.2)	193 (3.9)	258 (4.4)	227 (3.7)	228 (4.1)	202 (3.5)	209 (3.7)	273 (4.9)
**Heart attack (%)**										
No	4633 (95.4)	5140 (95.2)	4754 (94.6)	4752 (95.7)	5642 (95.2)	5939 (95.8)	5348 (96.3)	5534 (96.0)	5456 (95.6)	5288 (95.1)
Yes	221 (4.6)	257 (4.8)	273 (5.4)	215 (4.3)	282 (4.8)	261 (4.2)	203 (3.7)	230 (4.0)	251 (4.4)	270 (4.9)
**Liver condition (%)**										
No	4705 (96.9)	5239 (97.1)	4859 (96.6)	4801 (96.7)	5700 (96.2)	6011 (97.0)	5334 (96.1)	5524 (96.0)	5446 (95.5)	5260 (94.7)
Yes	153 (3.1)	158 (2.9)	169 (3.4)	163 (3.3)	226 (3.8)	189 (3.0)	219 (3.9)	233 (4.0)	258 (4.5)	293 (5.3)
**Chronic bronchitis (%)**										
No	4563 (93.9)	5101 (94.6)	4696 (93.4)	4672 (94.2)	5551 (93.7)	5890 (95.0)	5252 (94.6)	5439 (94.4)	5397 (94.7)	5167 (92.9)
Yes	299 (6.1)	291 (5.4)	330 (6.6)	290 (5.8)	375 (6.3)	311 (5.0)	297 (5.4)	320 (5.6)	304 (5.3)	395 (7.1)
**Emphysema (%)**										
No	4768 (98.0)	5305 (98.2)	4911 (97.7)	4868 (98.0)	5760 (97.2)	6076 (97.8)	5449 (98.2)	5669 (98.4)	5585 (97.8)	5449 (98.1)
Yes	95 (2.0)	96 (1.8)	117 (2.3)	98 (2.0)	164 (2.8)	137 (2.2)	100 (1.8)	95 (1.6)	125 (2.2)	106 (1.9)
**Current smoking status (%)**										
Non-smoker	2562 (52.7)	2800 (51.9)	2536 (50.4)	2623 (52.8)	3127 (52.7)	3351 (53.9)	3183 (57.3)	3236 (56.1)	3319 (58.2)	3233 (58.1)
Smoker	2295 (47.3)	2595 (48.1)	2496 (49.6)	2346 (47.2)	2802 (47.3)	2866 (46.1)	2369 (42.7)	2529 (43.9)	2386 (41.8)	2335 (41.9)
**BMI (mean (SD))**	28.38 (6.24)	28.20 (6.18)	28.41 (6.29)	28.77 (6.75)	28.96 (6.66)	29.16 (6.85)	28.78 (6.89)	29.10 (7.15)	29.54 (7.08)	29.85 (7.40)
**Age (mean (SD))**	50.52 (19.24)	49.98 (19.50)	50.84 (19.69)	48.33 (19.12)	50.77 (17.95)	49.61 (17.90)	48.94 (17.87)	49.11 (17.56)	49.53 (17.76)	51.50 (17.81)
**PIR (mean (SD))**	2.52 (1.60)	2.69 (1.63)	2.57 (1.59)	2.69 (1.59)	2.50 (1.61)	2.44 (1.62)	2.41 (1.67)	2.50 (1.65)	2.43 (1.60)	2.56 (1.60)

a: Data are unweighted

PIR: poverty-income ratio; BMI: body mass index

The percentages may not add up to 100% due to rounding

### Electronic supplementary material

Below is the link to the electronic supplementary material.

Supplementary Material 1: Supplementary data.

## Data Availability

The datasets used in the current study are available on the NHANES website: https://www.cdc.gov/nchs/nhanes/.
