# Supplementary material for: Trends of mental health care utilization among US adults from 1999 to 2018
Source: BMC Psychiatry. 2023 Sep 12;23:665. doi: 10.1186/s12888-023-05156-2 (PMC10496400; doi:10.1186/s12888-023-05156-2)
Supplement: Supplementary file 1 — Supplementary Material 1: Supplementary data. [file 12888_2023_5156_MOESM1_ESM.docx]

**Table S1. Total and gender specific percentage of mental health care utilization in US population, 1999-2018.**

| **Cycle** | **Total** |  |  | **Male** |  |  | **Female** |  |  |
| --- | --- | --- | --- | --- | --- | --- | --- | --- | --- |
|  | **Percentage ^a^** | **LCI** | **UCI** | **Percentage** | **LCI** | **UCI** | **Percentage** | **LCI** | **UCI** |
| 1999-2000 | 7.0 | 5.9 | 8.2 | 5.9 | 4.1 | 7.6 | 8.2 | 6.5 | 9.8 |
| 2001-2002 | 8.3 | 7.0 | 9.7 | 6.9 | 5.5 | 8.3 | 9.6 | 8.0 | 11.3 |
| 2003-2004 | 8.0 | 6.8 | 9.3 | 7.5 | 6.2 | 8.9 | 8.5 | 7.0 | 10.1 |
| 2005-2006 | 8.3 | 7.3 | 9.4 | 6.2 | 5.4 | 6.9 | 10.4 | 8.7 | 12.1 |
| 2007-2008 | 7.4 | 6.5 | 8.3 | 6.5 | 5.6 | 7.4 | 8.2 | 7.1 | 9.4 |
| 2009-2010 | 7.5 | 6.7 | 8.4 | 6.6 | 5.2 | 8.0 | 8.4 | 7.3 | 9.6 |
| 2011-2012 | 8.2 | 6.8 | 9.6 | 7.1 | 5.4 | 8.8 | 9.2 | 7.4 | 10.9 |
| 2013-2014 | 8.5 | 7.1 | 9.9 | 7.4 | 5.9 | 9.0 | 9.5 | 7.6 | 11.5 |
| 2015-2016 | 9.5 | 7.8 | 11.2 | 8.8 | 6.7 | 10.9 | 10.2 | 8.0 | 12.3 |
| 2017-2018 | 11.3 | 9.8 | 12.8 | 11.2 | 8.7 | 13.7 | 11.4 | 10.1 | 12.7 |

a: Weight, PSU and strata were applied to derive the population-representative estimation.

PSU: primary sample unit; LCI: lower confidential interval; UCI: upper confidential interval

**Table S2. Age-specific percentage of mental health care utilization in US population, 1999-2018.**

| **Cycle** | **20-39** |  |  | **40-59** |  |  | **60+** |  |  |
| --- | --- | --- | --- | --- | --- | --- | --- | --- | --- |
|  | **Percentage ^a^** | **LCI** | **UCI** | **Percentage** | **LCI** | **UCI** | **Percentage** | **LCI** | **UCI** |
| 1999-2000 | 7.0 | 5.5 | 8.4 | 8.5 | 6.4 | 10.5 | 5.1 | 3.7 | 6.5 |
| 2001-2002 | 8.5 | 6.4 | 10.6 | 9.9 | 8.6 | 11.2 | 5.1 | 3.6 | 6.7 |
| 2003-2004 | 7.8 | 6.1 | 9.6 | 10.6 | 8.9 | 12.4 | 4.0 | 2.7 | 5.4 |
| 2005-2006 | 7.5 | 6.2 | 8.8 | 11.1 | 9.5 | 12.6 | 5.2 | 3.9 | 6.5 |
| 2007-2008 | 8.4 | 7.2 | 9.6 | 9.1 | 7.3 | 10.8 | 3.2 | 2.3 | 4.0 |
| 2009-2010 | 8.9 | 7.8 | 10.0 | 8.4 | 6.5 | 10.2 | 4.2 | 2.8 | 5.5 |
| 2011-2012 | 9.7 | 6.8 | 12.5 | 8.5 | 6.6 | 10.4 | 5.7 | 3.8 | 7.5 |
| 2013-2014 | 9.6 | 8.3 | 10.9 | 9.5 | 6.8 | 12.2 | 5.6 | 4.4 | 6.9 |
| 2015-2016 | 11.3 | 8.4 | 14.2 | 9.9 | 7.9 | 12.0 | 6.7 | 4.8 | 8.5 |
| 2017-2018 | 13.5 | 11.2 | 15.9 | 12.2 | 9.6 | 14.9 | 7.4 | 5.7 | 9.2 |

a: Weight, PSU and strata were applied to derive the population-representative estimation.

PSU: primary sample unit; LCI: lower confidential interval; UCI: upper confidential interval

**Table S3. Race/ethnicity -specific percentage of mental health care utilization in US population, 1999-2018.**

| **Cycle** | **NHW** | | | **NHB** | | | **Hispanic** | | | **Other non-Hispanic** | | |
| --- | --- | --- | --- | --- | --- | --- | --- | --- | --- | --- | --- | --- |
|  | **Percentage ^a^** | **LCI** | **UCI** | **Percentage** | **LCI** | **UCI** | **Percentage** | **LCI** | **UCI** | **Percentage** | **LCI** | **UCI** |
| 1999-2000 | 7.4 | 5.9 | 8.9 | 6.2 | 3.7 | 8.8 | 5.0 | 2.8 | 7.3 | 10.8 | 5.1 | 16.4 |
| 2001-2002 | 9.1 | 7.5 | 10.6 | 6.7 | 3.7 | 9.6 | 7.1 | 3.4 | 10.8 | 4.4 | 0.6 | 8.2 |
| 2003-2004 | 8.8 | 7.1 | 10.5 | 7.6 | 6.2 | 9.0 | 5.0 | 3.7 | 6.3 | 5.6 | 0.9 | 10.2 |
| 2005-2006 | 9.0 | 7.4 | 10.6 | 7.1 | 5.5 | 8.7 | 4.7 | 2.9 | 6.5 | 9.9 | 6.8 | 13.1 |
| 2007-2008 | 8.1 | 7.1 | 9.1 | 6.1 | 3.9 | 8.3 | 6.0 | 4.6 | 7.3 | 5.0 | 2.8 | 7.1 |
| 2009-2010 | 8.2 | 6.8 | 9.6 | 7.8 | 6.0 | 9.7 | 5.2 | 4.2 | 6.2 | 5.3 | 3.0 | 7.7 |
| 2011-2012 | 9.1 | 7.3 | 10.9 | 7.7 | 4.9 | 10.5 | 5.5 | 3.8 | 7.2 | 6.2 | 3.3 | 9.0 |
| 2013-2014 | 9.0 | 7.0 | 11.0 | 8.5 | 5.7 | 11.3 | 7.3 | 6.2 | 8.4 | 6.8 | 5.3 | 8.3 |
| 2015-2016 | 10.6 | 8.2 | 12.9 | 8.9 | 7.0 | 10.9 | 7.2 | 5.4 | 9.0 | 6.9 | 4.0 | 9.7 |
| 2017-2018 | 11.7 | 9.3 | 14.2 | 11.1 | 9.1 | 13.0 | 10.8 | 8.5 | 13.1 | 9.8 | 6.5 | 13.1 |

a: Weight, PSU and strata were applied to derive the population-representative estimation.

PSU: primary sample unit; LCI: lower confidential interval; UCI: upper confidential interval.

NHW: non-Hispanic white, NHB: non-Hispanic black.

**Table S4. Sensitivity analysis for trend of mental health care utilization in US population, 1999-2018.**

| **Characteristics ^a^** | **OR ^b^** | **LCI** | **UCI** | **P value** |
| --- | --- | --- | --- | --- |
| **Total** | 1.04 | 1.02 | 1.06 | 0.001 |
| **Age** |  |  |  |  |
| 20-39 | 1.07 | 1.04 | 1.10 | 0.000 |
| 40-59 | 1.01 | 0.98 | 1.04 | 0.526 |
| 60+ | 1.05 | 1.01 | 1.09 | 0.022 |
| **Gender** |  |  |  |  |
| Male | 1.07 | 1.03 | 1.10 | 0.000 |
| Female | 1.02 | 0.99 | 1.04 | 0.169 |
| **Race/ethnicity** |  |  |  |  |
| Non-Hispanic white | 1.03 | 1.01 | 1.06 | 0.017 |
| Non-Hispanic black | 1.05 | 1.01 | 1.09 | 0.019 |
| Hispanic | 1.06 | 1.01 | 1.12 | 0.015 |
| Non-Hispanic other | 1.04 | 0.96 | 1.11 | 0.338 |

a: Weight, PSU and strata were applied to derive the population-representative estimation.

b: Models were adjusted with age, race/ethnicity, gender, family PIR category, and educational level.

OR: odds ratio; PSU: primary sample unit; LCI: lower confidential interval; UCI: upper confidential interval; PIR: income-to-poverty ratio.

**Figure S1. Non-linear trend for mental health utilization in age- and race/ethnicity-stratified populations, 1999-2018. A-D.** Non-linear trend for mental health utilization in NHW, NHB, Hispanic and other non-Hispanic populations. P for non-linearity in NHW, NHB, Hispanic and other non-Hispanic populations are 0.0634, 0.0093, 0.1821, and 0.0063, respectively. **E-G.** Non-linear trend for mental health utilization in populations stratified by age. P for non-linearity in populations aged 20-39, 40-59 and 60+ are all <0.0001. NHW: non-Hispanic white, NHB: non-Hispanic black.


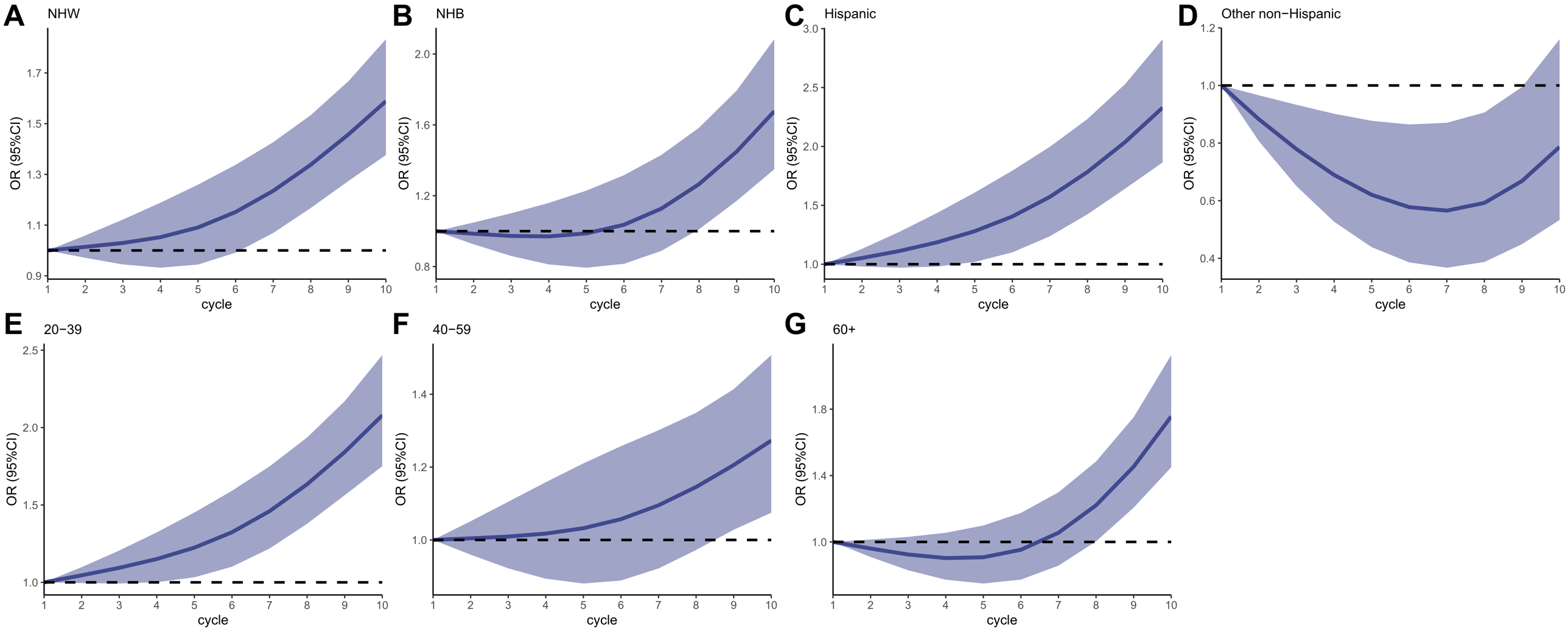
.
